# Influence of reproductive output on divorce rates in polar seabirds

**DOI:** 10.1002/ece3.7775

**Published:** 2021-09-12

**Authors:** Guillaume Mercier, Nigel G. Yoccoz, Sébastien Descamps

**Affiliations:** ^1^ Norwegian Polar Institute Fram Centre Tromsø Norway; ^2^ Department of Arctic and Marine Biology UiT The Arctic University of Norway Tromsø Norway

**Keywords:** Antarctica, breeding status, divorce, partner fidelity, Svalbard

## Abstract

The high occurrence of social monogamy in birds has led to questions about partner fidelity, or the perennial nature of monogamy from one breeding season to another. Despite the evolutionary advantages of partner fidelity, divorce occurs among 95% of bird species. We aimed to describe patterns of divorce and partner fidelity in five seabird species breeding in Arctic and Antarctic regions and investigated the influence of breeding status on pair bond maintenance. For four out of the five species considered, we observed low divorce rates (respectively 1.9%, 3.3%, 2.5%, and 0.0% for Brünnich's guillemot, glaucous gull, Antarctic petrel, and south polar skua), while the divorce rate was much higher (19.1%) for the black‐legged kittiwake. For kittiwakes, the divorce rate was lower for pairs that managed to raise their chick to 15 days of age, while the effect of breeding success on divorce in the four other species could not be tested due to the rareness of divorce events. Our results emphasize the potentially large temporal (interannual) variations that should be taken into account in understanding divorce and partner fidelity in seabirds.

## Graphical abstract

We studied divorce rates in five polar seabirds and found low divorce rates in four of them (respectively 1.9%, 3.3%, 2.5%, and 0.0% for Brünnich's guillemot, glaucous gull, Antarctic petrel, and south polar skua). Divorce rate was much higher (19.1%) for the black‐legged kittiwake and was dependent on previous breeding success. Our results emphasize the potentially large temporal (interannual) variations that should be taken into account in understanding divorce and partner fidelity in seabirds.

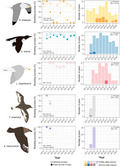

## 1 INTRODUCTION

The existence of monogamous mating systems in the animal kingdom has long been a topic of interest in evolutionary and behavioral ecology (Reichard, 2003). Defined as systems with an exclusive social relationship between one adult female and one adult male during a given reproductive event, socially monogamous mating systems are globally poorly represented in animals (Klug, 2018; Kvarnemo, 2018). They are almost absent in invertebrates (Mathews, 2002; McKeown & Shaw, 2008) and occur in few species of amphibians (Gillette et al., 2000; Tumulty et al., 2014), fish (Whiteman & Côte, 2004), and mammals (Lukas & Clutton‐Brock, 2013). However, with about 80% of species considered as socially monogamous, birds are exceptions to these global patterns (Black, 1996; Cockburn, 2006).

Pair bonds in birds have been described as typical examples of cooperative behavior in action (Black, 1996; Cockburn, 2006). Social monogamy has been shown to be favored in systems where the sharing of parental care within a pair (egg incubation, chick feeding, and defense), the lack of ability for individuals to sustain multiple partners during a breeding season (in terms of resources and territory) and the occurrence of active defensive behaviors to maintain unique access to a single partner occur (Brotherton & Komers, 2003; Gowaty, 1996; Grønstøl, 2018; Klug, 2018; Møller, 2003). Social monogamy is nevertheless not a monolithic term and finds one of its sources of variation in pair bond duration (Gowaty, 1996). The reunion of the two individuals forming a pair from one breeding event to another, called partner fidelity or perennial monogamy, occurs heterogeneously in monogamous bird species (Black, 1996; Griffith, 2019). While the ending of a pair bond may logically be induced by the death of one partner (widowing), it can also happen through a divorce when two birds forming a couple are still alive and pair with a new partner. Divorce has been recorded in 95% of socially monogamous bird species (Black, 1996; Choudhury, 1995; Culina et al., 2015; Ens et al., 1996).

From an evolutionary perspective, the maintenance of a pair bond throughout the years may be advantageous in terms of familiarity between mates, and enhancing the coordination and cooperation of the two members of a couple (Black, 1996; Bried & Jouventin, 2002; Choudhury, 1995; Sánchez‐Macouzet et al., 2014). Remaining faithful to the same partner also allows individuals to save energetic resources that would otherwise be allocated to obtaining a new mate without the risk of missing a breeding season (Bried & Jouventin, 2002). On the other hand, when a pair bond results in poor breeding success, the fitness benefits of divorce may exceed those of partner fidelity if divorce allows partner(s) to find a better mate and potentially achieve higher breeding success (Black, 1996; Bried & Jouventin, 2002; Choudhury, 1995; McNamara & Forslund, 1996). Therefore, divorce can be considered as an adaptive mechanism to correct for suboptimal partnerships associated with poor reproductive performance, with breeding success being a proxy used by birds to assess their partner's quality. The effect of breeding status on partner fidelity was analyzed in two meta‐analyses, based on 35 and 64 bird species, respectively, and an overall significant pattern of pairs with low breeding success having higher divorce rates was found (Culina et al., 2015; Dubois & Cézilly, 2002).

Besides these general patterns observed in meta‐analyses, responses can vary greatly among species, depending on life‐history traits such as longevity (Dubois et al., 1998; Ens et al., 1996; Jeschke & Kokko, 2008). Long‐lived species can indeed capitalize on mate fidelity and take advantage of the familiarity effect within a pair bond (Bried et al., 2003; Bried & Jouventin, 2002; Jeschke & Kokko, 2008) so that divorce is expected to be less beneficial for long‐lived species. The ecology of birds can also potentially add other constraints on partner fidelity and divorce patterns. Seabirds, for example, rely on marine resources for their food supplies but breed on land, so they must often travel considerable distances to find food for their chicks. In such systems, sharing parental care and coordination between partners is an important condition for successful reproduction and the choice of the reproductive partner is crucial (Bried & Jouventin, 2002). For birds breeding at high latitudes, the very short breeding season and the need to fulfill the breeding cycle in a shorter period of time can add another constraint on divorce and favor partner fidelity. Indeed, searching for a new mate may delay the initiation of breeding activities, which could be very costly in polar environments where breeding phenology is an important determinant of breeding success (Burr et al., 2016; Ens et al., 1996; Ritz et al., 2005). Furthermore, the familiarity between faithful mates may also be an important factor to achieve a successful breeding and raise offspring in these harsh and stochastic environments (Halimubieke et al., 2020).

The aim of this present study was to describe partner fidelity and divorce patterns of five seabird species breeding in polar environments (Arctic and Antarctic). We predicted low divorce rates for these long‐lived species breeding at high latitudes. We then investigated the influence of breeding success on divorce, testing the prediction that divorce should be higher following a breeding failure.

## 2 MATERIAL AND METHODS

### 2.1 Study system and species

**FIGURE 1:**
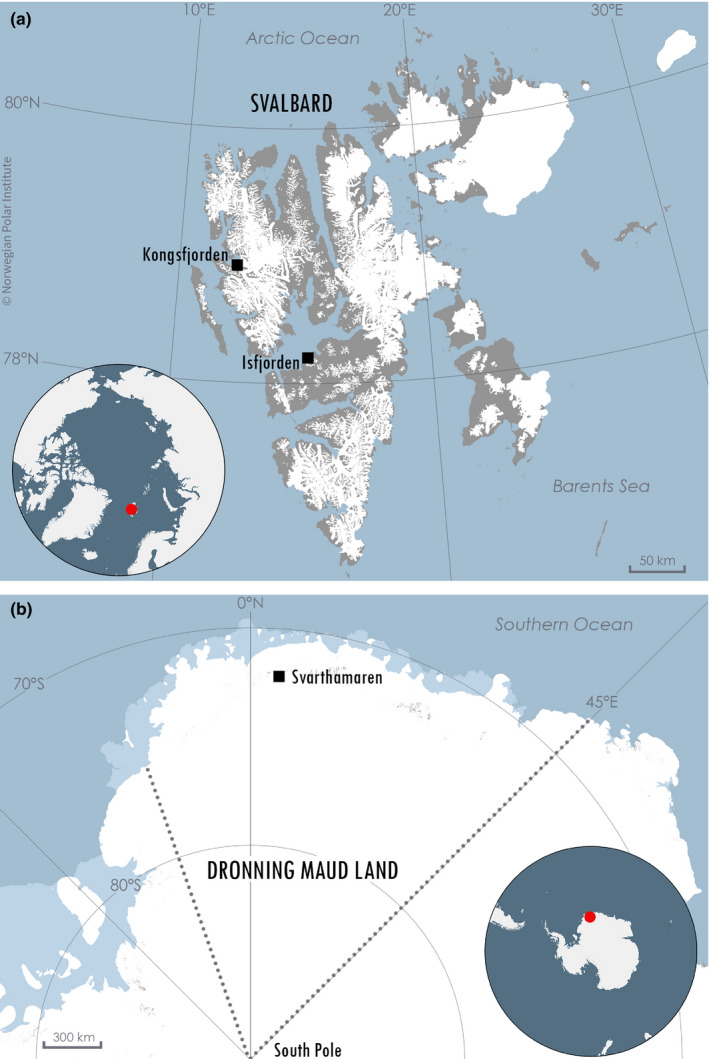
Study sites in (a) Svalbard, Arctic and (b) Dronning Maud Land, Antarctica. Colonies in Svalbard were located in two fjords: Isfjorden (78°18′N, 15°10′E) for black‐legged kittiwakes and Kongsfjorden (78°56′N,12°25′E) for Brünnich's guillemot and glaucous gull. Svarthamaren (71°53′S, 5°10′E) in Dronning Maud Land was located ca. 200 km inland and was used as a nesting site by the south polar skua and the Antarctic petrel

We based our study on five seabird species: the black‐legged kittiwake (*Rissa tridactyla*), the Brünnich's guillemot (*Uria* *lomvia*), the glaucous gull (*Larus hyperboreus*), the Antarctic petrel (*Thalassoica antarctica*), and the south polar skua (*Stercorarius maccormicki*). Data from the first three species were from colonies in two fjords of the high Arctic Svalbard Archipelago (Figure 1). The kittiwake colony was located in Isfjorden (Grumantbyen) at an abandoned human settlement where buildings are used as a nesting ground for approximately 45 pairs. Situated in Kongsfjorden, the Brünnich's guillemot colony (Ossian Sarsfjellet) consisted of a bird cliff used as a nesting ground by approximately 1,000 pairs. Also breeding in Kongsfjorden, glaucous gull nests were distributed across the entire fjord (ca. 100 nests in total in the fjord). The two other species, the Antarctic petrel and the south polar skua, were breeding at Svarthamaren, an ice‐free area (nunatak) located ca. 200 km inland in Dronning Maud Land, Antarctica. With between 100,000 and 250,000 breeding pairs, this colony is one of the largest Antarctic petrel colonies (Descamps et al., 2016; Schwaller et al., 2018; Van Franeker et al., 1999). Svarthamaren also hosts 100–150 skua breeding pairs nesting in the lower flat parts, relying exclusively on petrel eggs and chicks as their food resource during the breeding season (Busdieker et al., 2020).

These five species are seabirds, which breed on land and remain at sea during the internuptial period (Coulson, 2011; del Hoyo et al., 1992; Delord et al., 2020; Frederiksen et al., 2012, 2016; Gaston & Jones, 1998; Weimerskirch et al., 2015; Weiser & Gilchrist, 2020). They are all characterized by high adult survival rates and thus long lifespans (adult survival rate: 0.85 for *R. tridactyla*, 0.88 for *U. lomvia*, 0.85 for *L. hyperboreus*, 0.91 for *T. antarctica* and 0.91 for *S. maccormicki*, Anker‐Nilssen et al., 2020; Descamps et al., 2016; Fluhr et al., 2017; S. Descamps, unpublished data). Clutch size varies between species but is low (1 egg for Brünnich's guillemot and Antarctic petrel, 1–2 eggs for black‐legged kittiwake breeding on Svalbard, 1–2 eggs for south polar skuas, and 1–3 eggs for glaucous gulls). The amount of time individuals allocate to chick parental care fluctuates between ca. two weeks for the Brünnich's guillemot (for female parental care only as males stay longer with their chick), 5 weeks for the south polar skua, 6 weeks for the Antarctic petrel, 5–7 weeks for the black legged kittiwakes, and up to seven weeks for the glaucous gull (del Hoyo et al., 1992). These five species are socially monogamous and partners share breeding duties (nest building, egg brooding, and parental care) until the departure from the colony at the end of the breeding season (del Hoyo et al., 1992), except for Brünnich's guillemot, for which males stay longer with the chicks after they leave the nest at an age of 15–30 days (Gaston & Hipfner, 2020; Young et al., 2013). Males and females may have different wintering strategies (e.g., Bogdanova et al., 2011 for *R. tridactyla*; Frederiksen et al., 2016 for *U. lomvia*), but the consequences on the pair bond are unknown. The high average resighting rates of all species in the study sites indicate high site fidelity and low emigration rates (average resighting rates obtained from capture–mark–recapture data modeling: 0.73 for *L. hyperboreus* and *S. maccormicki*, 0.74 for *R*. *tridactyla,* 0.79 for *T. antarctica,* and a 0.90 for *U*. *lomvia;* unpublished data).

### 2.2 Divorce data

Colonies were monitored from 3 to 11 years from 2009 to 2020 (Table 1). Fieldwork and data collection took place annually from early or mid‐incubation to mid‐ or late chick rearing (early/mid‐June to the end of July/beginning of August in Svalbard and from early December to mid‐February at Svarthamaren). Every year, a sample of breeding adults nesting at each colony were caught with a nylon loop attached to a telescopic pole while at the nest and ringed with a metal ring and a coded plastic ring for identification at a distance. Blood or feathers were sampled to determine the sex of the individuals using molecular analyses (details about the sexing procedure in Harris et al., 2020 and Tarroux et al., 2020). Colonies were visited regularly during the breeding season (every two to four days on average), and during each visit, the identity of birds occupying the nests and the breeding status (number of eggs or chicks, hatching of eggs) were recorded. These observations allowed the determination of the breeding pairs and their breeding status for a sample of nests every year (Table 1). Several visits may have been necessary to identify breeding pairs, which led to annual average breeding success being potentially over‐estimated (as pairs failing very early in the season were less likely to be identified and thus to be included in the study). Accordingly, pair bond status from one year to the next was determined and classified as (a) fidelity when two individuals of a pair at year *t* were observed at least once in the same nest in year *t* + 1; (b) divorce when two individuals of a pair in year *t* were still alive (observed at the colony) but not forming a pair during year *t* + 1. Furthermore, the hatching success (HS) and chick survival 15 days after hatching (CS15d) were used to reflect the early and late breeding success of a pair, respectively. Due to the difficulty in monitoring the survival of glaucous gull chicks after hatching, only hatching success was used for this species. Ultimately, only pairs for which we were able to retrieve breeding and pair bond status in years *t* and *t* + 1 were used for testing the effect of divorce on breeding success (Table 1).

**TABLE 1:** Study period and number of pairs whose status and breeding success were identified for each species (D, divorce; Fi, fidelity; NA, status not used for the analyses)

	*Rissa tridactyla*	*Uria lomvia*	*Larus hyperboreus*	*Stercorarius maccormicki*	*Thalassoica antarctica*
Study period	2009–2020	2011–2020	2011–2020	2011–2014	2011–2014
Years of monitoring	11	9	9	3	3
Number of couples with known pair bond status	118 (76 Fi, 18 D, 24 NA)	175 (152 Fi, 3 D, 20 NA)	32 (29 Fi, 1 D, 2 NA)	43 (38 Fi, 5 NA)	46 (40 Fi, 1 D, 5 NA)
Number of couples with known pair bond and breeding status	89	146	29	37	25

### 2.3 Statistical analyses

#### 2.3.1 Influence of breeding status on pair bond status

Statistical analyses were carried out with R software (R Core Team, 2019) using generalized linear mixed models under a Bayesian framework with the *rstanarm* package (Gelman et al., 2020). Our initial goal was to test for a relationship between divorce and breeding success for all five species, but due to the very low number of divorces in most species (see results), we could only test this for the black‐legged kittiwake. Partner fidelity from year *t* to *t* + 1 (response variable) was modeled as a binary variable (0 = fidelity, 1 = divorce) with a binomial distribution and a logit link function. Three variables (fixed effects) reflecting the different stages of breeding were alternatively used to test the effect of breeding status on the response variable: the hatching status (HS, failure, or success), the chick survival 15 days after hatching (CS15d, failure, or success), and the overall breeding (OB, failure or success), that is, the product of HS and CS15d. The breeding success or failure was defined by the presence or absence of at least one egg or chick at the nest. Three other variables were included in the models as random effects, as they structure the study design and potentially influence the response variable. They consist of the year, used as a proxy of average annual abiotic (environmental conditions, climatic fluctuations) and biotic (predation, prey availability) pressures (Botero & Rubenstein, 2012; Christensen‐Dalsgaard et al., 2018; Descamps et al., 2015, 2016), the pair identity, assumed to reflect the intrinsic quality of the pairs (Bried & Jouventin, 2002) and the nest identity, illustrating the potential quality difference among nesting places across the colony (Massaro et al., 2001; Varpe & Tveraa, 2005). In our systems, even though a given pair often used the same nest, this was not always the case and both variables were not equivalent. Models computed were of the following form: *logit* *(pair bond status_t + 1_) = α + β × breeding status_t_ + (1|year_t_) + (1|nest_t_) + (1|couple_t_)*, with α corresponding to the intercept (global mean response), β the breeding status effect (HS or CS15d or OB), and 1|year*_t_*, 1|nest*_t_*_,_ and 1|couple*_t_* the random effects. We used the stan_glmer function with five chains of 30,000 iterations for each model (Stan Development Team, 2020). Default weakly informative prior distribution for the Bayesian model was implemented to reduce posterior uncertainty and stabilize computations (Muth et al., 2018). Convergence of chains was assessed following the procedure described by Muth et al. (2018) using the *shinystan* function (Stan Development Team, 2017). The leave‐one‐out cross‐validation (hereafter, LOOIC), an information criterion adapted to a mixed models approach, was used to compare models and determine their relative goodness of fit (Vehtari et al., 2017).

## 3 RESULTS

### 3.1 Pair bond and breeding status observations

**FIGURE 2:**
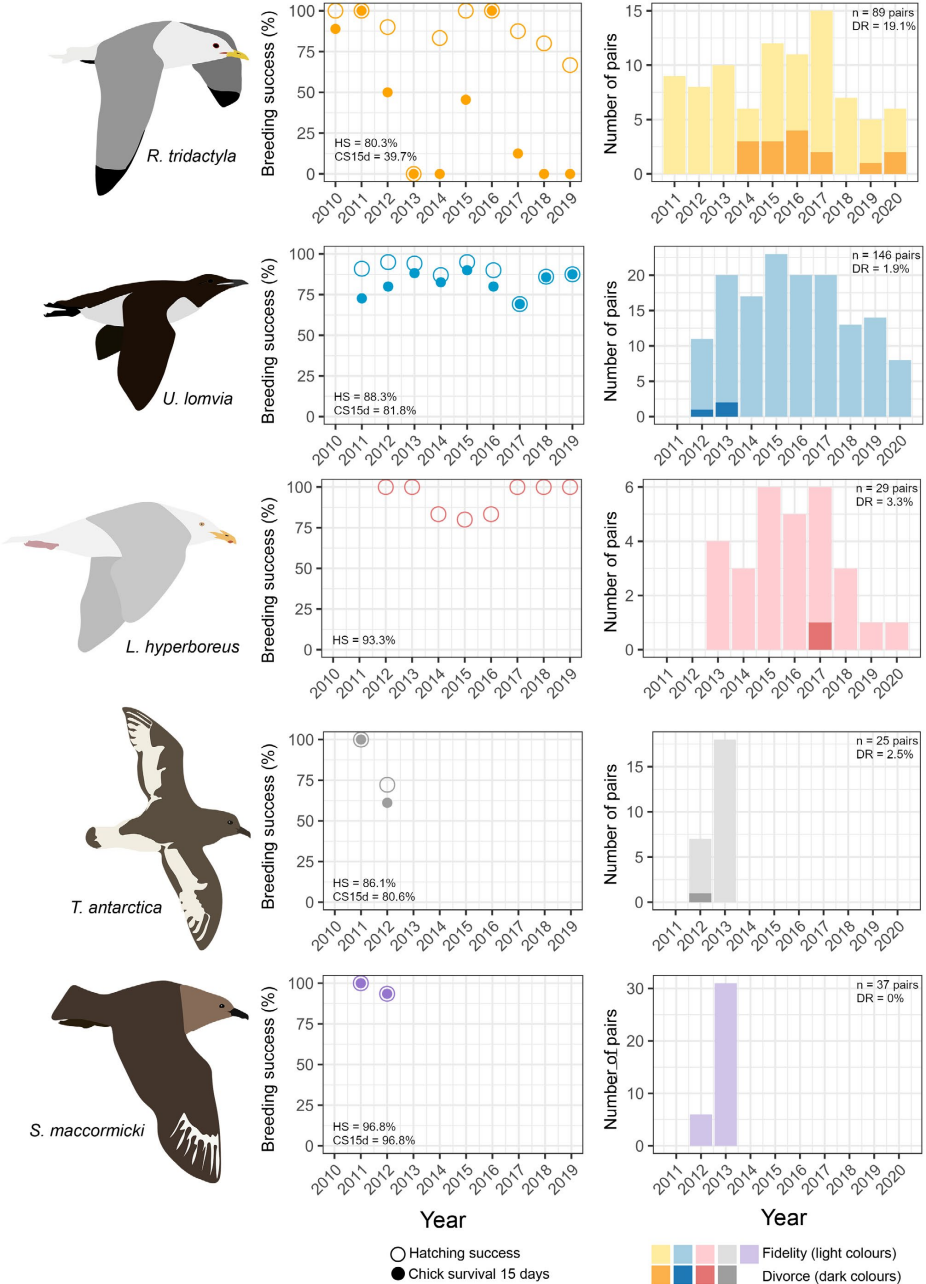
Breeding success and pair bond status for five polar seabirds. Left panels: breeding success with average hatching success (HS, open circles) and chick survival 15 days after hatching (CS15d, closed circles). Right panels: number of pairs with known bond and breeding status (*n*: total number of pairs and DR: average divorce rate). DR was calculated using all individuals with known pair bond status (including those with unknown breeding status, see Table 1). Light colors represent the number of pairs remaining faithful, while dark colors represent the number of pairs that divorced. Influence of breeding status on divorce

Breeding success of black legged kittiwakes showed large interannual variability with a total breeding failure in 2013 (Figure 2). Divorce rates varied among years and divorces were observed in five out of the 11 study years (2014, 2015, 2016, 2017, 2019, 2020; Figure 2). Kittiwake divorce rates ranged from 13.3% to 50% and was equal to 19.1% for the studied population (Figure 2). For the two other species where long‐term data were available, Brünnich's guillemot and the glaucous gull, interannual variability in breeding success was low (Figure 2). Hatching success averaged 87% and 93% for these two species, respectively, and 82% of the chicks survived up to 15 days after hatching for Brünnich's guillemot. These two species experienced low divorce rates over the study period (1.9% for Brünnich's guillemot, 3.3% for glaucous gull; Figure 2). Breeding success of the Antarctic petrel varied during the two years of monitoring and only one divorce was observed for this species, leading to a divorce rate of 2.5% (Figure 2). For the south polar skua, breeding success was generally high (96.6% in average) and no divorce was observed during the study period (among 43 pairs; Figure 2).

**FIGURE 3:**
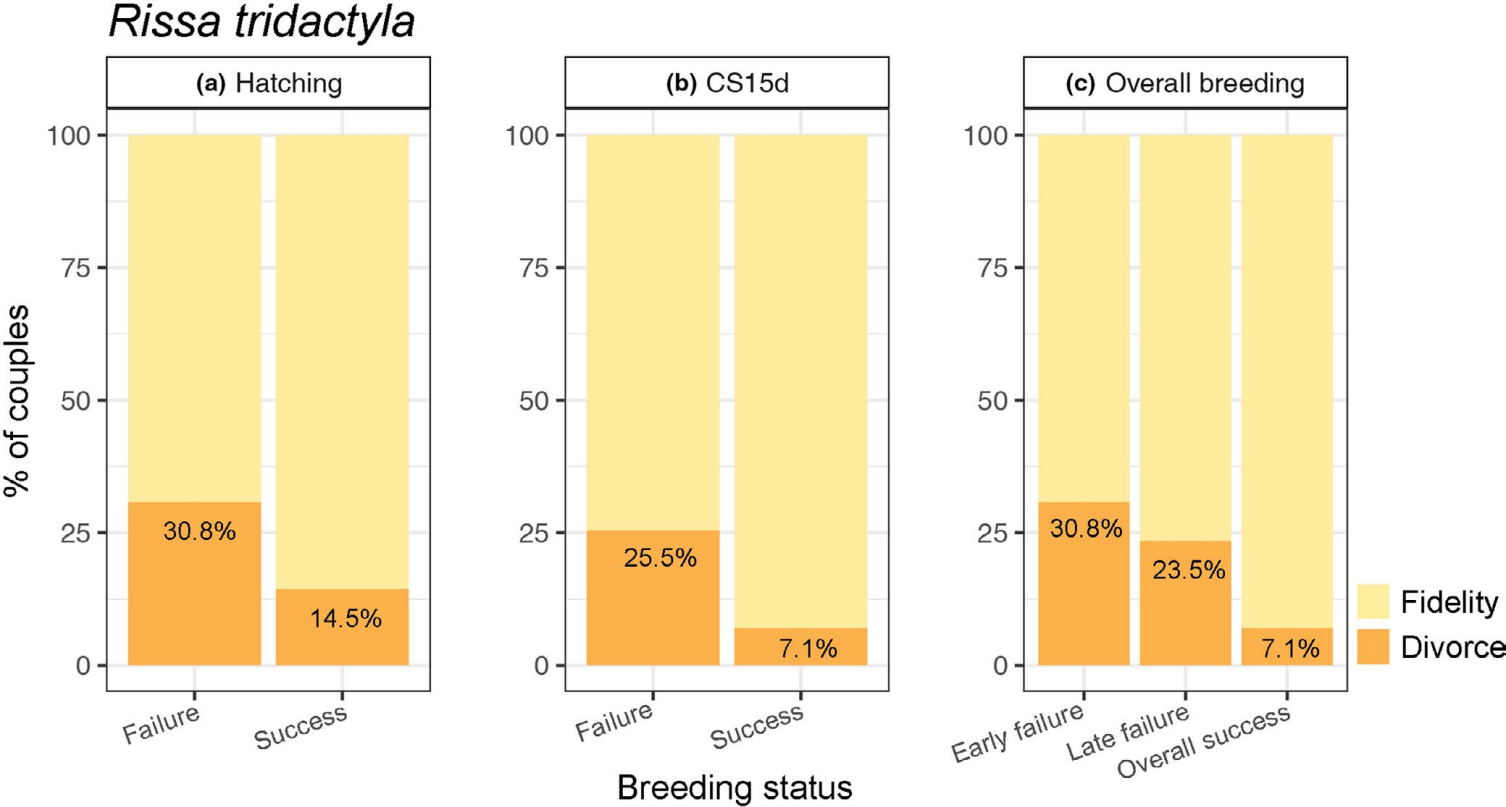
Influence of breeding status on divorce rates for the black‐legged kittiwake (*Rissa tridactyla*). Different variables reporting breeding status of birds are presented in the three panels, hatching status (left panel), CS15d (chick survival 15 days after hatching, middle panel), and the overall breeding (early failure: hatching failure, late failure: hatching success but death of the chick(s) within the first 15 days, overall success: hatching success and chick survival 15 days after hatching; right panel)

For black‐legged kittiwakes, the proportion of couples divorcing after a breeding failure was higher than after a breeding success (30.8% vs. 7.1% overall breeding; Figure 3). Furthermore, the probability for a breeding pair to divorce decreased the later the breeding failure occurred (30.8% divorce for early breeding failure, 25.5% for late breeding failure, Figure 3). These results were partly supported by our model selection (Table 2). Indeed, the model with the lowest LOOIC included the chick survival variable (CS15d; Table 2) but the difference in LOOIC with the null model was small (ΔLOOIC=0.6). For the Antarctic petrels and glaucous gulls, the only divorce was observed for a pair that was successful in the previous year. For Brünnich's guillemot, two of the three divorces occurred after a successful breeding.

**TABLE 2:** Output of the generalized linear mixed models testing the effect of breeding status on divorce for Svalbard black‐legged kittiwakes

Model	Number of parameters	Variables (fixed and random)	Estimates	*SD*/*SE*	LOOIC	Δ LOOIC
Null model	3	Intercept	1.9	0.6 *SE*	80.5	0.6
		1|couple		1.0 *SD*		
		1|nest		0.9 *SD*		
		1|year		1.5 *SD*		
HS	4	Intercept	1.3	1.2 *SE*	82.5	2.6
		HS	0.8	1.2 *SE*		
		1|couple		1.2 *SD*		
		1|nest		0.9 *SD*		
		1|year		1.4 *SD*		
**CS15d**	**4**	**Intercept**	**1.3**	**0.6 *SE***	**79.9**	**0.0**
		**CS15d**	**1.8**	**1.0 *SE***		
		**1|couple**		**1.0 *SD***		
		**1|nest**		**0.8 *SD***		
		**1|year**		**1.1 *SD***		
OB	4	Intercept	−0.3	1.4 *SE*	80.6	0.7
		OB	1.0	0.6 *SE*		
		1|couple		1.1 *SD*		
		1|nest		0.9 *SD*		
		1|year		1.1 *SD*		

The model in bold is the one with the lowest LOOIC.

## 4 DISCUSSION

The high occurrence of social monogamy in birds in comparison to other animals has raised questions about partner fidelity and divorce. In our study, based on five seabird species breeding in polar regions, we assessed divorce occurrences and their connection with previous breeding success. For four out of the five species considered, we observed very low divorce rates (respectively 1.9%, 3.3%, 2.5%, and 0.0% for Brünnich's guillemot, glaucous gull, Antarctic petrel, and south polar skua) while divorce was much higher (19.1%) for the black‐legged kittiwake and negatively associated with previous breeding success.

### 4.1 Divorce and partner fidelity in seabirds

For Brünnich's guillemot and the Antarctic petrel, our results constitute the first numerical values of divorce rate and partner fidelity. In Brünnich's guillemot, partner fidelity has been suggested to be very high, and thus, the divorce rate very low, as observed in other Alcid species sharing a range of ecology and life‐history traits (divorce rates of 9.2%–11.7% for Common guillemots (*Uria aalge*); 5.7% for razorbills (*Alca torda*); and 5.7%‐16.0% for Atlantic puffins (*Fratercula arctica*)*;* Ashcroft, 1979; Ens et al., 1996; Gaston & Hipfner, 2020; Harris & Wanless, 1989). For Procellariforms, to which the Antarctic petrel belongs, generally low divorce rates were also found (median divorce rate of 8.8% for 31 species, Bried et al., 2003). For the three other species, the divorce rates obtained in this study were in line with those observed previously, despite some intraspecific variation (see details below). Indeed, the average divorce rate of the kittiwake on Svalbard (19.1%, this study) was similar those observed in Alaska (19.3%, Hatch et al., 1993), France (26.0%, Naves et al., 2006), and the UK (26.1%, Coulson & Thomas, 1980). In another Svalbard colony (Kongsfjorden), based on 32 pairs over three years, a much higher divorce rate (45.7%) was observed (Angelier et al., 2007). Concerning the glaucous gull, one previous study in Canada had estimated their divorce rate at 9% (Gaston et al., 2009), which is higher but in the same order of magnitude as our observations on Svalbard (3%). For the south polar skuas, the fact that no divorce occurred during our study contrasts with some results obtained earlier (divorce rates of 1.5%, 9.1%, and 15%, Ainley et al., 1990; Bried & Jouventin, 2002; Pietz & Parmelee, 1994). The differences in monitoring effort and numbers of couples identified (e.g., 23 years of monitoring in a colony of 1,000 breeding pairs and a significant ringing effort for Ainley et al., 1990; 34 couples over four breeding seasons for Pietz & Parmelee, 1994) may explain these variations. A possible underestimation of divorce rates in our study might also explain the differences observed compared to other studies. Indeed, in our approach, a couple was described as divorced when both partners were observed alive at the colony. However, it is possible that in some cases, one of these partners had not been observed at the colony even if present, which could thus have led to an underestimation of the total number of divorces.

Among our studied species, divorce was thus generally low and there was a general tendency to maintain pair bonds through time. This corresponds to general observations in seabirds and in birds in general. Indeed, when comparing our results to the 209 species of monogamous birds for which we were able to retrieve a measure of partner fidelity in the literature, we observed that divorce occurs in 95% of the species, and half of the divorce rates were below 15% (Appendix S1). Large differences between species are, however, observed, ranging from birds repairing with a new partner every season (100% divorce, e.g., great blue and gray herons [*Ardea herodias*] and [*Ardea cinerea*], common house martin [*Delichon urbicum*]) to strict partner fidelity (0% divorce, e.g., Eurasian nuthatch [*Sitta europaea*], and common pigeon [*Columba livia,* Appendix S1]). Similar patterns were observed for seabirds specifically, with a median divorce rate of 13.8% (Appendix S1). While phylogeny can be an important driver of these evolutionary patterns, different life‐history and ecological traits emerge in the literature to explain interspecies variations such as longevity (Jeschke & Kokko, 2008), degree of coloniality (Dubois et al., 1998), or whether birds are migratory or resident (Ens et al., 1996). Among our study species, all colonial breeders and migratory birds, a lower adult survival rate (85%) was observed for the black‐legged kittiwake, compared to 88 to 91% for the Brünnich's guillemot, Antarctic petrel, and south polar skua, which could partly explain their higher divorce rate (Anker‐Nilssen et al., 2020; Descamps et al., 2016; Fluhr et al., 2017; unpublished data). However, the adult survival of Svalbard glaucous gull was the same as kittiwake and its divorce rate was much lower as well (unpublished data). This indicates that the among‐species variation in divorce and partner fidelity likely depends on a suite of life‐history traits (Culina et al., 2015) and cannot be explained by the species longevity alone.

Genetic monogamy may be important to consider in understanding divorce rates and its among‐species variation. Beyond socially monogamous mating systems, birds can indeed be involved in extrapair paternity (Griffith et al., 2002). Such situations could allow individuals to maintain a socially monogamous pair bond, leading then to low divorce rates in the population, while increasing their reproductive values by mating with other partners. However, extrapair paternities are likely rare for our studied species and represent only a very small amount of the chicks produced (7.0% for *S. maccormicki* and *T. antarctica*, Griffith et al., 2002; 0% for *R. tridactyla*, Helfenstein et al., 2004; low occurrence in Larid and Alcid species, Anker‐Nilssen et al., 2008, 2010).

### 4.2 Temporal and spatial variations in divorce rates

Divorce may be dependent on environmental variations and vary both spatially and temporally. Indeed, in the course of our study, we observed important interannual variations in divorce rate for the species for which we had long‐term data (9–11 years for Arctic species). While these variations in divorce rates may represent random changes due to small sample sizes, they could also be attributed to fluctuation in yearly environmental conditions (e.g., weather, Botero & Rubenstein, 2012) or population structure (e.g., sex ratio, Liker et al., 2014). Such interannual variations could explain the differences in divorce rates observed for some species among the different studies (e.g., 19.1% divorce for kittiwakes in our study vs. 45.7% in Angelier et al., 2007). This emphasizes the importance of considering multiple years and ideally multiple sites when studying divorce or partner fidelity in a given species, and divorce rates estimated from a single year and/or single colony (including the divorce rates estimated for Antarctic petrel and south polar skua in our study) should be interpreted with caution.

The breeding environment can also constitute an important factor for bird partnerships. At high latitudes, the phenological window to fulfill breeding duties is shorter, which can eventually affect mate fidelity patterns (Bried & Jouventin, 2002; Burr et al., 2016) and, more specifically, may increase the fitness costs of divorce. Indeed, in polar environments, reproduction must be initiated early enough to produce chicks during the short summers and delaying the onset of breeding may lower breeding success (Descamps, 2019; Descamps et al., 2011; Dunn, 2004; Sauve et al., 2019). As looking for a new partner may take time and delay reproduction (Ens et al., 1996), it may thus entail higher fitness costs at high latitudes as compared to nonpolar environments. This hypothesis could, at least partly, explain why we observed so few divorce events in four of the study species (Brünnich's guillemot, glaucous gull, south polar skua, Antarctic petrel) but additional studies are needed to confirm this.

### 4.3 Does breeding failure affect the probability to divorce?

Breeding success is expected to affect divorce rates in birds and the probability of a divorce should increase following a breeding failure (Culina et al., 2015; Dubois & Cézilly, 2002). For black‐legged kittiwakes, divorce is a physiologically and energetically costly process (Angelier et al., 2007; Chardine, 1987). Due to the lack of coordination in a newly formed couple, higher baseline corticosterone levels, indicating prolonged stress levels, have been measured for divorced individuals (Angelier et al., 2007). Moreover, newly formed couples undergo a diminution of time off‐duty and an augmentation of reproductive effort (increase in greeting ceremonies within the couple, longer copulations) (Chardine, 1987). Such an increase in reproductive effort occurs at the cost of other activities (e.g., feeding activities, maintenance of body conditions), which may ultimately impact individual fitness. Accordingly, our results indicated that the divorce rate was 17% higher following a breeding failure (i.e., failed hatching or death of the chick within the first 15 days) than following a breeding success in Svalbard black‐legged kittiwakes, though the statistical support for such an increase was not strong. This increase was relatively low compared to other studies on black‐legged kittiwakes that showed an increase of 34% (Cap Sizun, France, Naves, 2005) and 32% (Shiefields, UK, Coulson, 1972) in divorce rate after a breeding failure. The low size of our study colony (45 breeding pairs) possibly restricted the probability of repairing after divorce and consequently inhibited divorce mechanisms (i.e., the fitness cost of divorcing would be too high considering the low probability to find a new partner). The breeding failures observed in this colony may also have been caused by different factors (e.g., nest falling from the ledge, predation by glaucous gulls, starvation), not all necessarily associated with bird quality. It may thus be important to assess the drivers of breeding failures to better understand the relationships between breeding output and divorce. Finally, the age‐structure in our study colony may also differ from those in these other kittiwake colonies (Cap Sizun, France, Naves, 2005 and Shiefields, UK, Coulson, 1972). Age is known to affect breeding success and divorce (Ens et al., 1996), but was unfortunately unknown for the individuals included in our study.

## 5 CONCLUSION

Our results complement previous studies by studying mating systems and partner fidelity for species in polar environments. While divorce was rare in four of the species studied, it was more frequent for black‐legged kittiwakes and was higher after a breeding failure. Moreover, we found large interannual variations in pair bond status, stressing the importance of multi‐year studies and the caution needed when interpreting results from short‐term studies. In Arctic and Antarctic regions, the environment is rapidly changing (Barros et al., 2014; van der Bilt et al., 2019), but what this means for partner fidelity and divorce patterns remains to be elucidated.

## CONFLICT OF INTEREST

The authors declare no competing interests.

## AUTHOR CONTRIBUTIONS

**Guillaume Mercier:** Conceptualization (equal); Formal analysis (lead); Methodology (equal); Writing‐original draft (lead). **Nigel G. Yoccoz:** Formal analysis (equal); Methodology (equal); Supervision (supporting); Validation (equal); Writing‐review & editing (supporting). **Sébastien Descamps:** Conceptualization (lead); Funding acquisition (lead); Project administration (lead); Supervision (lead); Writing‐original draft (supporting); Writing‐review & editing (equal).

## Supporting information

Appendix S1

## Data Availability

The data associated with this manuscript are available from the Norwegian Polar Institute Datacenter (https://data.npolar.no/dataset/d506b9fa‐5bf4‐4b51‐8f83‐1589018ec311, https://doi.org/10.21334/npolar.2021.d506b9fa).
